# Advances in Proximity-Assisted Bioconjugation

**DOI:** 10.1021/acs.accounts.5c00368

**Published:** 2025-09-25

**Authors:** Mary Canzano, Gonçalo J. L. Bernardes

**Affiliations:** ‡ Yusuf Hamied Department of Chemistry, 2152 University of Cambridge, CB2 1EW, Cambridge U.K.; δ Translational Chemical Biology Group, Spanish National Cancer Research Centre (CNIO), Madrid 28029, Spain

## Abstract

Proximity-induced chemistry (PIC) refers to the transient reactivity between two or more molecules upon physical closeness which are otherwise unreactive. Harnessed by nature to control fundamental biological processes such as transcription and signal transduction, PIC increases the probability of correctly oriented, effective collisions, facilitating fundamental cellular processes. Within the field of chemical biology, PIC has been employed for several clinically relevant purposes, including the degradation of aberrant biomolecules and construction of protein therapeutics. This Account focuses on the application of PIC strategies for the development of site-specific bioconjugation techniques, termed proximity-assisted bioconjugation (PAB). Site-specific bioconjugation refers to the precise modification of biomolecules to generate homogeneous products. Such techniques are vital for the development of protein therapeutics including antibody–drug conjugates (ADCs), the investigation of the biological mechanisms of post-translational modifications (PTMs), and the visualization of biomolecular interactions *in vitro* and *in vivo*. While numerous strategies have been developed, many suffer from poor yields, limited product stability, demanding experimental procedures, and/or a lack of regioselectivity. Thus, PIC principles have been implemented to address these limitations, leading to the development of PAB strategies which achieve precise, regioselective modification of biomolecules. In this Account, we describe the development of PAB techniques within our group at the University of Cambridge and Instituto de Medicina Molecular (iMM) over the past five years. Our journey with PAB began serendipitously while investigating maleic acid derivatives for cysteine bioconjugation. Here, we discovered the secondary participation of proximal lysines on Trastuzumab-V205C and Gemtuzumab-V205C, conjugatable THIOMAB antibodies commonly used in ADCs, leading to the formation of distinct bioconjugate products relative to IgGs without such lysines. Further investigation into the proximal lysine (K207) of Trastuzumab-V205C revealed that residue 207 could be harnessed directly or mutated to precisely tune the stability of ADCs due to proximity interactions between K207 and covalent modifications of C205. Considering that two Trastuzumab drug conjugates are approved for clinical use, these findings have contributed to the evolving understanding of the chemical landscape of this antibody and help inform future ADC design and development. Further, we describe efforts from our group to develop two distinct PAB approaches: regioselective lysine acetylation of histone H3 and phage display-compatible peptide cyclization. These strategies combine induced-proximity with traditional bioconjugation techniques to enable regioselective modification of biomolecules which are historically difficult to selectively modify. These methods are readily adaptable to related systems and serve as representative examples of how to successfully develop PAB strategies for desired applications. In short, this Account highlights our group’s contributions to and insights on PAB methodologies wherein we illustrate how PIC can be thoughtfully applied to bioconjugation techniques for various aims including regioselective bioconjugation and enhanced bioconjugate stability. We expect that PAB approaches will continue to diversify bioconjugation applications and greatly expand the toolkit of chemical biologists.

## Graphical abstract

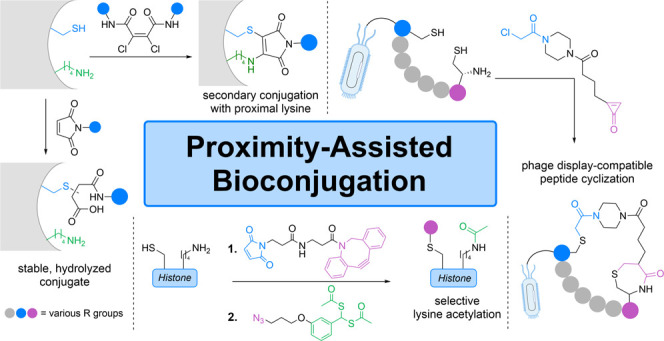

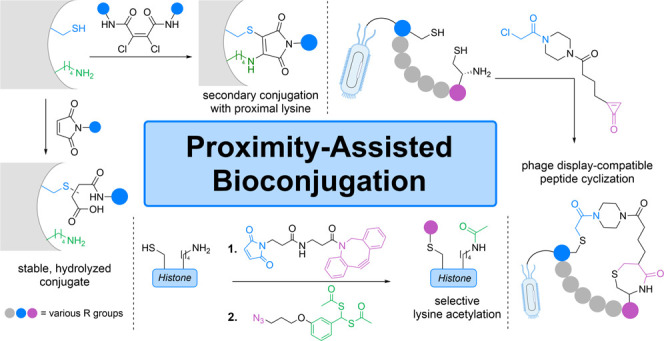

## Key References

-

Laserna, V. ; Abegg, D. ; Afonso, C. F. ; Martin, E. M. ; Adibekian, A. ; Ravn, P. ; Corzana, F. ; Bernardes, G. J. L. Dichloro Butenediamides as Irreversible Site-Selective Protein Conjugation Reagent. Angew. Chem., Int. Ed. 2021, 60 (44), 23750–23755 10.1002/anie.202108791PMC859679034472678.^1^ We developed cysteine-reactive reagents which demonstrate secondary conjugation with proximal lysine residues on several proteins with specific chemical features.
-

Ferhati, X. ; Jiménez-Moreno, E. ; Hoyt, E. A. ; Salluce, G. ; Cabeza-Cabrerizo, M. ; Navo, C. D. ; Compañón, I. ; Akkapeddi, P. ; Matos, M. J. ; Salaverri, N. ; Garrido, P. ; Martínez, A. ; Laserna, V. ; Murray, T. V. ; Jiménez-Osés, G. ; Ravn, P. ; Bernardes, G. J. L. ; Corzana, F. Single Mutation on Trastuzumab Modulates the Stability of Antibody-Drug Conjugates Built Using Acetal-Based Linkers and Thiol-Maleimide Chemistry. J. Am. Chem. Soc. 2022, 144 (12), 5284–5294 35293206 10.1021/jacs.1c07675PMC8972253.^2^ This work investigates the impact of proximal Lys207 of Trastuzumab-V205C in stabilizing conjugates produced with maleimides and destabilizing acetal-based linkages.
-

Afonso, C. F. ; Marques, M. C. ; António, J. P. M. ; Cordeiro, C. ; Gois, P. M. P. ; Cal, P. M. S. D. ; Bernardes, G. J. L. Cysteine-Assisted Click-Chemistry for Proximity-Driven, Site-Specific Acetylation of Histones. Angew. Chem., Int. Ed. 2022, 61 (46), e202208543 10.1002/anie.202208543PMC982850036124857.^3^ Here, we established a PAB technique to generate homogeneous, regioselectively acetylated lysine conjugates of histone H3 through the introduction and subsequent modification of a proximal cysteine.
-

Brown, L. ; Vidal, A. V. ; Dias, A. L. ; Rodrigues, T. ; Sigurdardottir, A. ; Journeaux, T. ; O’Brien, S. ; Murray, T. V. ; Ravn, P. ; Papworth, M. ; Bernardes, G. J. L. Proximity-Driven Site-Specific Cyclization of Phage-Displayed Peptides. Nat. Commun. 2024, 15 (1), 7308 39181880 10.1038/s41467-024-51610-4PMC11344848.^4^ In this work, we developed a new PAB strategy to cyclize peptides for phage display using cyclopropenone chloroacetamide (CCA).

## Introduction

Proximity-induced chemistry (PIC) refers to the unique reactivity of two or more species upon spatial proximity that are otherwise unreactive. In nature, PIC is employed ubiquitously to precisely, selectively, and temporally control various cellular processes including signal transduction and enzyme catalysis.^5^ The utility of PIC can be illustrated by the advantageous features of a simple intramolecular reaction: the probability of correctly oriented, effective collisions is exponentially increased by the inherent proximity invoked by the presence of two reactive groups on the same molecule.^6^

In recent years, several PIC strategies have been developed to address persistent challenges in chemical biology including therapeutic strategies for diseases modulated by ‘undruggable’ proteins and facile approaches to construct homogeneous, stable ADCs.^5,7^ Broadly, PIC can be divided into two related subfields within chemical biology: inducing proximity to modulate biomolecular interactions and to alter the reactivity of functional groups in biological systems.

### Inducing Biomolecular Interactions

The advent of molecular glues in 1991 and proteolysis targeting chimeras (PROTACs) in 2001 revealed the utility of PIC in targeting biomolecules which were previously thought to be “undruggable”.^8,9^ This class of molecules brings two or more species (e.g., proteins, RNA, small molecules, etc.) together to induce specific phenotypic responses. PROTACs promote the formation of an intermediate ternary complex with a protein-of-interest (POI) and E3 ligase to trigger ubiquitination of the POI, targeting it to the proteasome for degradation (Figure 1a). Various PROTACs have been designed to degrade aberrant proteins associated with disease pathologies including KRAS^G12C^,^10^ Bruton’s tyrosine kinase (BTK) mutants,^11^ and STING.^12^ Additional PIC degraders have been developed including lysosome-targeting chimeras (LYTACs) which direct proteins to the lysosome for degradation (Figure 1b)^13^ and ribonuclease-targeting chimeras (RIBOTACs) for RNA degradation (Figure 1c).^14^

**Figure 1:**
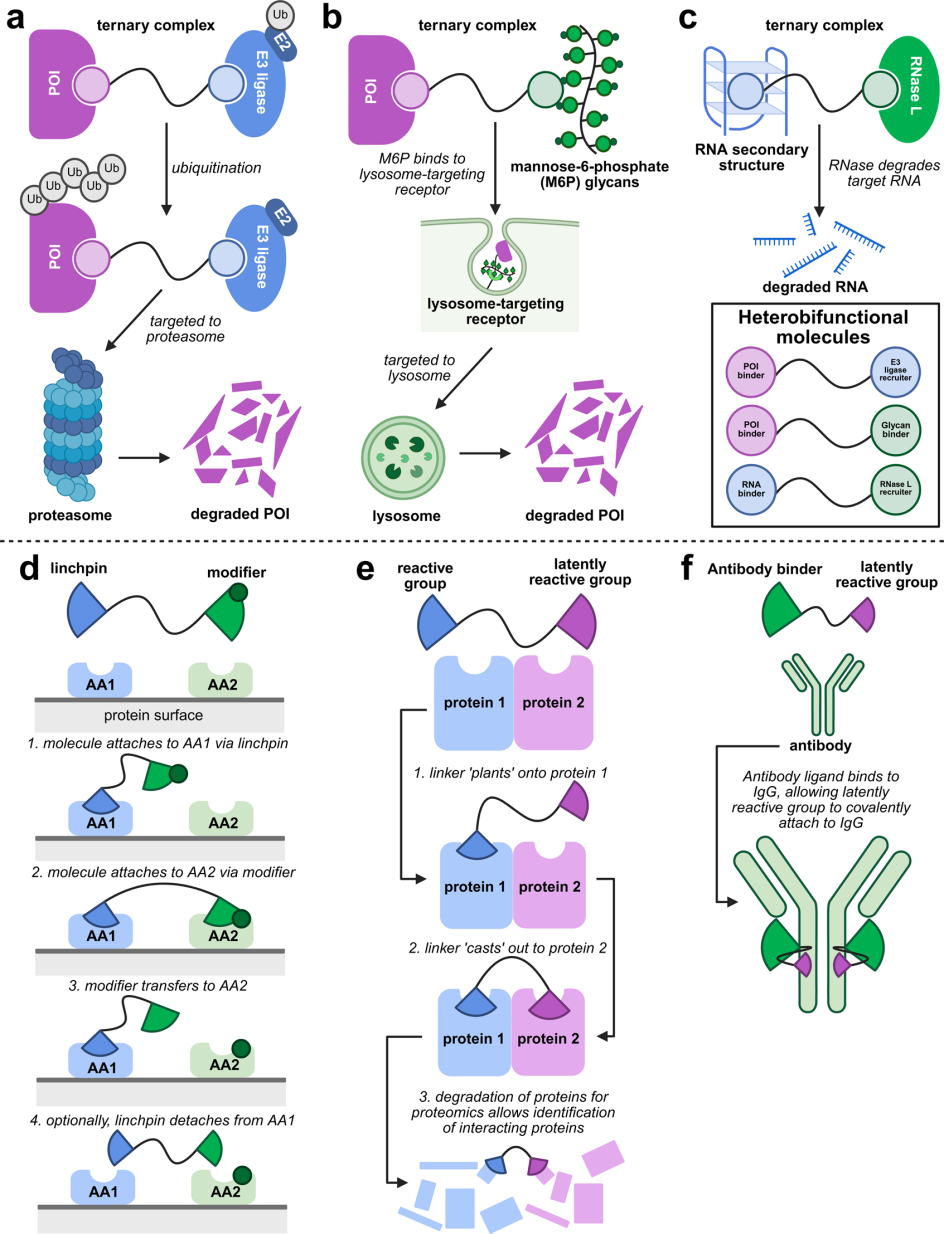
Proximity-induced strategies in chemical biology involving modulation of biomolecular interactions (a–c) and of functional group reactivity (d–f). Mechanisms of PROTACs (a), LYTACs (b), RIBOTACs (c), linchpin-directed modification (d),^22^ plant-and-cast cross-linking (e),^23^ and proximity-induced antibody bioconjugation (f).^24^

Beyond degraders, this subfield has diverse applications for basic science, translational, and clinical research. For example, techniques to discern the biological mechanisms of PTMs have been developed using heterobifunctional molecules which recruit PTM writer and eraser enzymes to POIs.^15–17^ Additionally, several molecular glue therapeutics have been developed, including Tacrolimus (FK506), a potent immunosuppressant which crucially regulates immune system signal transduction by binding FKBP,^18^ and Acoramidis (AG10) which binds and stabilizes transthyretin to treat transthyretin-mediated amyloid cardiomyopathies.^19^

### Modulating Functional Group Reactivity

Nature frequently employs proximity to modulate the reactivity of functional groups for diverse purposes. For example, while serine, histidine, and aspartate are relatively inert amino acids, they gain unique functionality upon formation of the Ser-His-Asp catalytic triad in the active site of serine proteases.^20,21^ Thus, to enable selective reactions on biomolecules, many have tried to emulate this approach through the development of PIC techniques that alter the reactivity of functional groups upon spatial proximity.

One such example is linchpin-directed modification which enables selective bioconjugation via the linchpin of a designed linker which covalently attaches to one residue and enables the site-specific transfer of a modifier to a nearby target residue (Figure 1d).^22^ Protein cross-linking via a ‘plant-and-cast’ approach and homogeneous antibody bioconjugation have also been achieved using latently reactive functional groups which selectively modify their target only upon the induced proximity from covalent attachment or ligand-IgG binding, respectively (Figure 1e–f).^23,24^ Additionally, several covalent therapeutics have been developed in this subfield, including antibodies which irreversibly cross-link their antigens to prevent tumor growth and engineered PD-1 (programmed cell death protein 1) which covalently anchors T cells to cancer cells upon binding its ligand.^7^

Over the years, our group has contributed to both subfields of PIC through the design of a PROTAC for APT1,^25^ the development of proximity-induced nucleic acid degraders (PINADs),^26^ recent work on enhancement of phagocytic synapses (ENPHASYS) to recruit macrophages to cancer cells,^27^ and various projects on proximity-assisted bioconjugation (PAB).^1–4^ This Account focuses on PAB as a novel, relatively unexplored topic, and we direct any interested readers to previous reviews from our group regarding PROTACs and RNA degraders.^28,29^

Site-specific bioconjugation is an indispensable tool in chemical biology which enables the production of homogeneous, post-translationally modified biomolecules.^30–32^ Strategies for protein bioconjugation include the chemical modification of canonical amino acids such as cysteine or lysine,^30^ genetic code expansion with ncAAs,^33^ enzyme-mediated ligation,^34^ and incorporation of protein tags.^35,36^ Despite a large toolkit of strategies, each technique has fundamental limitations; notably, many are challenging to implement, suffer from poor yields, have limited product stability, and/or are unable to achieve regioselectivity, the ability to specifically react with one residue over other residues of the same type (e.g., one specific lysine among many lysines). Thus, many have explored the use of PIC to expand the arsenal of bioconjugation techniques, particularly to achieve regioselectivity. Several PAB strategies have since been developed for numerous applications including for the production of ADCs, visualization of biomolecules *in vivo*, and investigation of PTM functions, firmly establishing the utility of PIC for bioconjugation.^22–24,37–53^

In this Account, we summarize our efforts to develop PAB strategies to address persistent challenges in chemical biology. Beginning with a story about maleic acid derivatives as potential bioconjugation reagents, we describe the incidental discovery of PAB wherein proximal lysines on several proteins, including two antibodies commonly used to develop ADCs, participate in cysteine bioconjugation. Later, an investigation into the proximal lysine of Trastuzumab-V205C revealed that residue lysine 207 (K207) directly modulates the stability of C205 conjugates via proximity effects. This finding enabled the precise tuning of ADC stability and greatly contributes to the overall understanding of the molecular architecture of this prominent IgG.

Motivated by the clear utility of PAB, we also designed two distinct PAB strategies to regioselectively acetylate histone lysines to study the biological mechanisms of PTMs and to cyclize peptides for phage display without compromising phage viability or infectivity. Both strategies leverage latently reactive functional groups to achieve regioselective bioconjugation upon induced proximity, yielding biochemically validated histone conjugates and novel cyclic peptide binders for streptavidin, respectively. Through a synopsis of our efforts to develop PAB techniques, we reveal the utility of PIC to improve upon existing bioconjugation strategies and facilitate modification of challenging targets. Furthermore, our contributions may serve as a guide to others in the development of new PAB methods to address specific biological problems.

## Participation of Proximal Residues in Bioconjugation

The relevance of PIC for bioconjugation was a serendipitous finding in a project led by Dr. Victor Laserna (University of Cambridge) that investigated maleic acid derivatives for cysteine bioconjugation. Among canonical amino acids, cysteine is valued for the exceptional nucleophilicity of its thiolate side chain, making it a common target for bioconjugation with electrophiles such as maleimides, carbonylacrylic reagents, and iodoacetamides (Figure 2a). Cysteine favorably has a low natural abundance in the proteome (<2%),^55^ and, when present, is commonly buried in structural disulfides.^56^ Therefore, selective modification of proteins can often be achieved by introducing a single free cysteine to proteins via site-directed mutagenesis.

**Figure 2:**
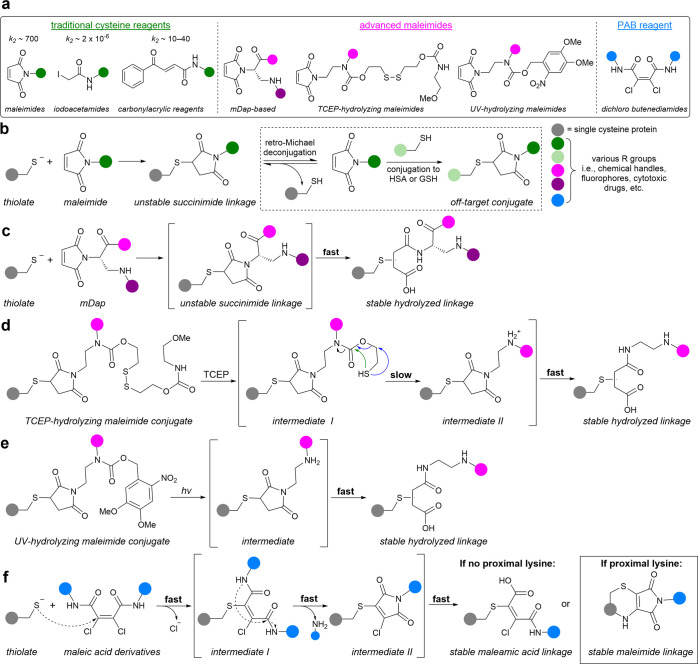
Strategies for cysteine bioconjugation. (a) Categories of cysteine-reactive reagents. Conjugation rate constants reported in M^–1^ s^–1^.^57,62,63^ (b) Conventional cysteine-maleimide Michael addition is susceptible to deconjugation and subsequent conjugation of maleimides to other reactive thiols (dashed box). (c) mDap maleimides promote hydrolysis of the thio-succinimide linkage and form stable bioconjugates.^60^ (d–e) Bioconjugates made with TCEP- (d) or UV-hydrolyzing (e) maleimides enable controlled hydrolysis of thio-succinimide linkages.^61^ Colored arrows represent two possible mechanisms (d). (f) Novel PAB strategy to modify single-cysteine proteins with dichloro butenediamides.

Michael addition with maleimides is traditionally the methodology of choice for cysteine bioconjugation given the rapid kinetics^57^ and facile experimental conditions (room temperature compatibility, short reaction times, few equivalents, etc.).^58^ However, the susceptibility of the thio-succinimide linkage to retro-Michael deconjugation and subsequent thiol exchange is a significant drawback to cysteine-maleimide conjugation (Figure 2b). This is particularly relevant for ADC use *in vivo* where the free thiols of glutathione (GSH) and human serum albumin (HSA) can react with deconjugated maleimide linkers, reducing on-target cytotoxicity and introducing off-target toxicity.^59^ Accordingly, several strategies have been developed to prevent deconjugation using a proximal amine to hydrolyze the thio-succinimide linkage, including foundational work from Peter Senter and co-workers^60^ and recent efforts from our group which unmask a proximal amine upon disulfide reduction and self immolation or UV irradiation (Figure 2c–e).^61^ While these strategies have proven efficacious, in 2021, we were interested in maleic acid derivatives as potential alternatives to maleimides, in hopes of achieving similar conjugation kinetics without requiring extra synthetic design to achieve product stability.

Through screening various maleic acid derivatives, Dr. Victor Laserna and colleagues identified dichloro butenediamides as robust, cysteine-reactive reagents.^1^ Under mild reaction conditions, cysteine was rapidly and irreversibly modified via a maleamic acid linkage (Figure 3a). Mechanistically, Michael addition of the cysteine to the alkene was followed by rapid cyclization to a maleimide derivative via intramolecular attack of the amide to the distal carbonyl (Figure 2f). This cyclic intermediate could be observed briefly by LC–MS before undergoing rapid hydrolysis to a stable, linear maleamic acid linkage. Importantly, dichloro butenediamides exhibited comparable kinetics to maleimide reagents, as demonstrated by ∼1:1 product formation upon treating proteins with equimolar **1** and *N*-benzyl maleimide, and bioconjugates were not susceptible to deconjugation in the presence of other reactive thiols (1 mM glutathione or ∼5% human plasma).

**Figure 3:**
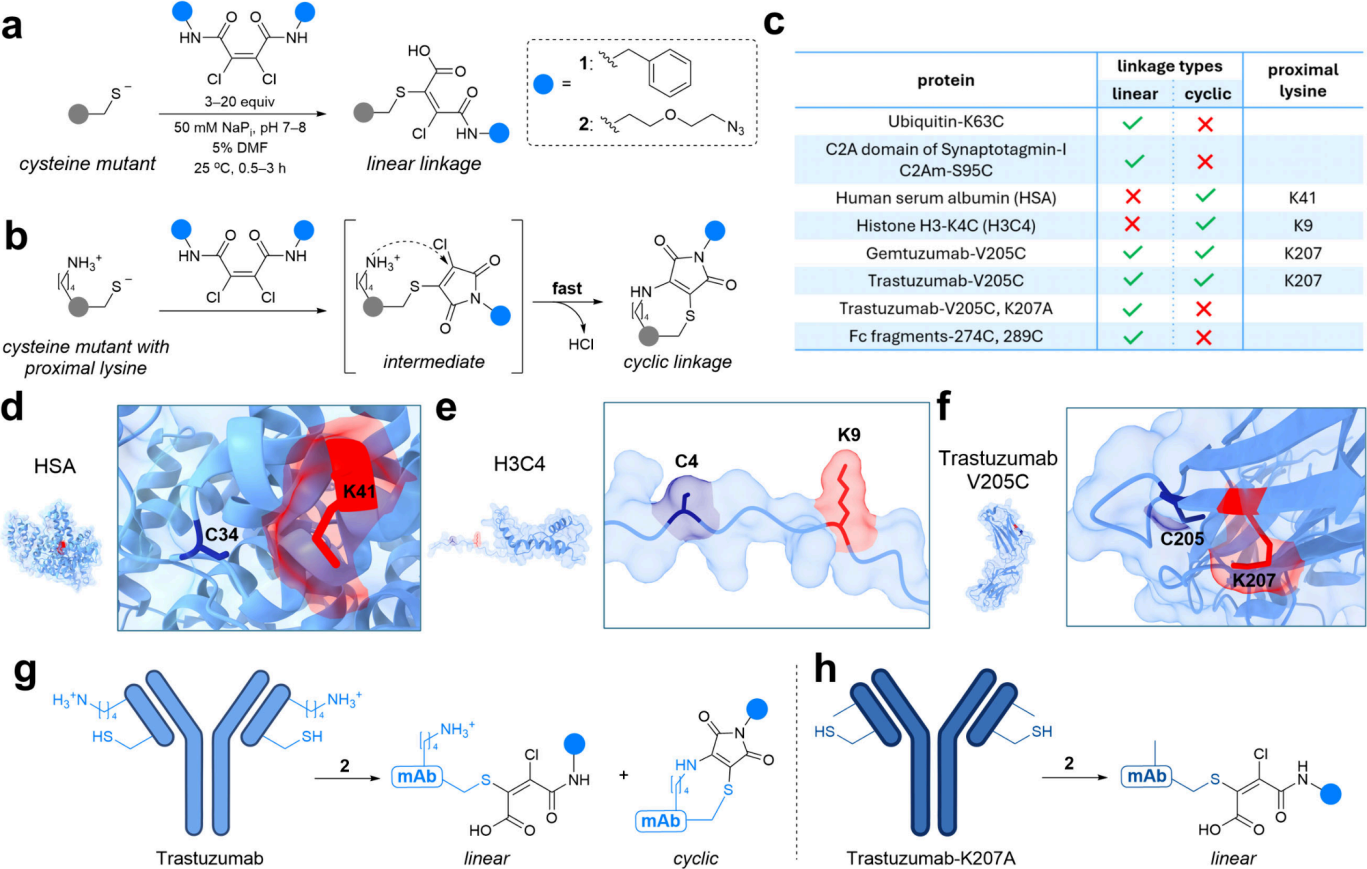
Dichloro butenediamides form secondary linkages with proteins containing proximal lysines. (a, b) Modification of single-cysteine proteins without (a) or with (b) proximal lysines. (c) Linkage types for protein library screened with **2**. (d–f) Space filling models of the proteins which exhibit cyclic linkages: HSA (d), H3C4 (e), and Trastuzumab-V205C. Cysteine colored in dark blue and proximal lysine in red. Proteins were visualized in ChimeraX. (g, h) Bioconjugation of Trastuzumab (g) or Trastuzumab-K207A (h) with **2** yields both linkages and only the linear linkage, respectively. mAb represents Trastuzumab and has been visualized as a single chain for simplification.

However, upon screening **2** with numerous proteins (Figure 3c), LC–MS data revealed the formation of a distinct product with a mass 54 Da less than expected for certain proteins, corresponding to the loss of H_2_O and HCl. Notably, both HSA and Histone H3 K4C (H3C4), formed this alternative product (Figure 3b, c, d, and e). Since these proteins contain a free cysteine with a lysine nearby, we suspected that upon formation of the maleimide intermediate on cysteine, nucleophilic attack by the proximal lysine yields a stable, cyclic linkage corresponding to the observed mass (Figure 3b). While lysines primarily exhibit reactivity with maleimides under basic conditions,^64^ this reactivity between these species near neutral pH appeared to be proximity-driven.

Interestingly, when THIOMABs Gemtuzumab-V205C and Trastuzumab-V205C were treated with **2**, both linkages were observed, with the cyclic form predominating (Figure 3c, g). Thus, although not previously reported, we suspected the presence of a lysine proximal to C205 on these IgGs that participates in bioconjugation. To investigate this, in collaboration with Professor Francisco Corzana at the Universidad de La Rioja, molecular dynamics (MD) simulations of the Fab fragment of Trastuzumab-**2** identified Lys207 (K207) as the lysine most proximal to the reactive carbon (C–Cl) (Figure 3f). Experimentally, MS/MS studies of Trastuzumab-**2** confirmed that the cyclic linkage contained both C205 and K207, providing conclusive validation of the computational findings. Further, bioconjugate Trastuzumab-K207A-**2**, in which the proximal lysine was mutated to alanine, exhibited only the linear linkage, indicating that K207 is required for cyclic linkage formation (Figure 3h).

Presently, Trastuzumab remains a popular candidate for ADC development, with two conjugates approved for clinical use.^65,66^ Thus, this finding that proximal K207 can participate in conjugation reactions with C205 was intriguing and has contributed to the collective understanding of the molecular architectures of C205 THIOMABs. Although there was no apparent benefit nor disadvantage to the cyclic linkage, as both linkage types exhibited no evidence of deconjugation in the presence of GSH at 37 °C for 66 h, we suspected that the behavior of K207 may differ with the use of other reagents and warranted further investigation.

## Tunable Stability of Antibody–Drug Conjugates (ADCs) with PAB

Driven by Xhenti Ferhati (Universidad de La Rioja), Ester Jiménez-Moreno (Universidad de La Rioja), and Emily Hoyt (University of Cambridge), our initial aim was to investigate acetal-based linkers for the development of Trastuzumab-V205C ADCs.^2^ The proximity effects of K207 quickly emerged as a significant factor for these bioconjugates, and in continued collaboration with the Corzana group, investigating the local chemical environment of this IgG became our primary focus.

Acetals are prone to hydrolysis in acidic conditions but demonstrate improved stability at neutral pH,^67^ providing a potential mechanism to reduce off-target drug toxicity by triggering drug release in the acidic conditions of the tumor microenvironment (pH 5.6–7)^68^ or upon internalization in the endosome (pH 5.0–6.5) and lysosome (pH 4.5–5.0).^69^ While this technique has been well established for hydrazone-based linkers,^70^ at the time, it had largely been unexplored for acetals with a few notable exceptions.^71,72^

To test this approach, we synthesized three acetal-based linkers with a cysteine-reactive handle and a duocarmycin prodrug (**4**–**6**). Duocarmycin is a cytotoxic DNA alkylating agent which can be activated from its prodrug through acid-catalyzed acetal hydrolysis and subsequent spiro cyclization (Figure 4a, b).^73,74^ Linker **3** was also synthesized with a masked fluorophore to assess the efficacy of acetal hydrolysis. Bioconjugate Trastuzumab-**3** quickly established proof-of-concept, as it released the fluorophore under acidic conditions and demonstrated stability at neutral pH (50% vs 15% hydrolysis at pH 5 vs pH 7.2 over 24 h at 37 °C), demonstrating comparable behavior to hydrazone-based linkers (20–100% vs 0–10% hydrolysis at pH 4.5 vs neutral pH depending on exact substituents) (Figure 4c, e).^75^

**Figure 4:**
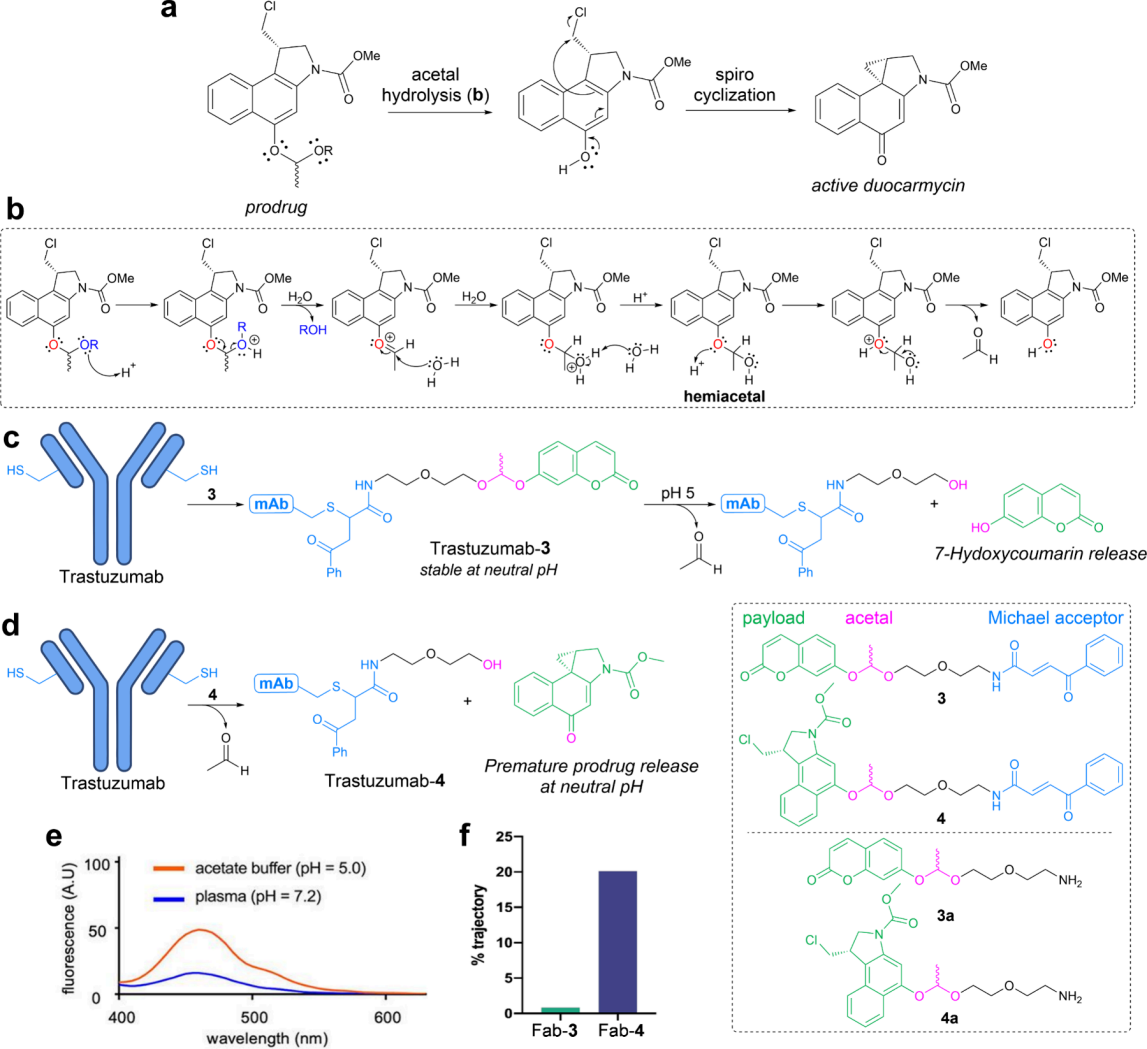
Evaluation of acetal-based linkers for pH-mediated ADCs. (a, b) Mechanism for duocarmycin prodrug release. Arrow pushing mechanism for acid-catalyzed acetal hydrolysis seen in (b). (c) Trastuzumab-**3** is stable at neutral pH and releases 7-hydroxycoumarin under mildly acidic conditions. (d) Trastuzumab-**4** is hydrolyzed as it forms, causing premature prodrug release. (e) Fluorescence emission of Trastuzumab-**3** upon incubation for 24 h at different pHs. (f) Comparison of Fab-**3** and Fab-**4**. Percentage of total trajectory time (500 ns) where distance between Nε–C (of acetal) is ≤ 4.5 Å. Panels e and f adapted from (^2^). Copyright 2022 American Chemical Society.

Surprisingly, when **4** was conjugated to Trastuzumab, the intended product could not be produced. Acetal hydrolysis occurred under several tested bioconjugation conditions; at best, ∼50% conversion to intact Trastuzumab-**4** was achieved (Figure 4d). This was an unexpected finding considering that **4** was stable under the reaction conditions, and no such difficulties occurred when conjugating with **3**. However, informed by our findings on K207 with maleic acid derivatives, we suspected a proximity effect might be involved.

To explore this, MD simulations were performed on the Fab fragments of Trastuzumab conjugates, revealing transient hydrogen bonds between the acetal oxygens and the ammonium group of K207 for Fab-**4**. Interestingly, no such contacts were found for Fab-**3**, despite the same protein sequence and acetal linkage (Figure 4f). However, predicted logP values for **3a** and **4a** (1.0 vs 2.4, respectively) indicate that **4** is more hydrophobic and thus more likely to be positioned closer to the protein’s surface. Therefore, we suspected that the proximity of K207 to the acetal of anchored **4** enables the lysine to act as an acid catalyst.

We confirmed this experimentally by eliminating the proximity effect of K207 via two approaches. First, substitution of lysine for alanine greatly improved conjugate stability, with Trastuzumab-K207A-**4** demonstrating only ∼20% acetal hydrolysis over 24 h at neutral pH compared to ∼40% for Trastuzumab-**4** (Figure 5b, g). Second, decreasing the proximity between K207 and the acetal by utilizing a longer linker (**5**) was highly effective. MD simulations comparing Fab-**4** and Fab-**5** revealed a significant reduction in the proximity between the lysine and acetal for Fab-**5** (∼8 Å change) (Figure 6). Experimentally, neither Trastuzumab-**5** nor Trastuzumab-K207A-**5** conjugates demonstrated any evidence of hydrolysis over 4 h at 37 °C at neutral pH, revealing enhanced acetal stability when proximity effects are diminished (Figure 5c, d, and g).

**Figure 5:**
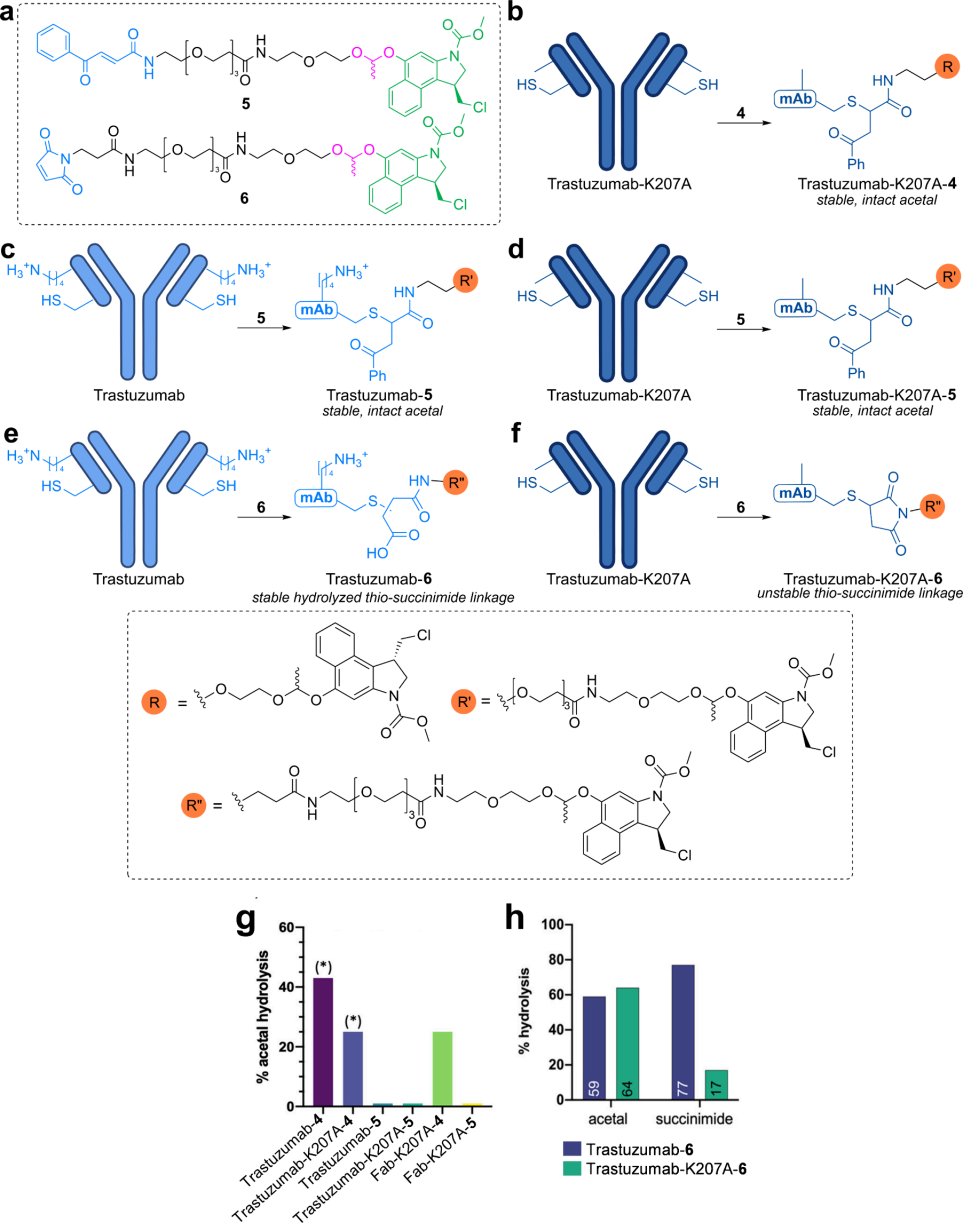
Optimizing the stability of IgG conjugates. (a) Linkers **5** and **6**. (b–f) Generation of Trastuzumab-K207A-**4** (b), Trastuzumab-**5** (c), Trastuzumab-K207A-**5** (d), Trastuzumab-**6** (e), and Trastuzumab-K207A-**6** (f). (g) Percent acetal hydrolysis via LC–MS following incubation at pH 7, 37 °C, 4 h, or (*) pH 7, 25 °C, 24 h. (h) Percent acetal and succinimide hydrolysis of **6** conjugates via LC–MS following incubation at pH 7.2, 37 °C, 24 h. Panels g and h adapted from (^2^). Copyright 2022 American Chemical Society.

**Figure 6:**
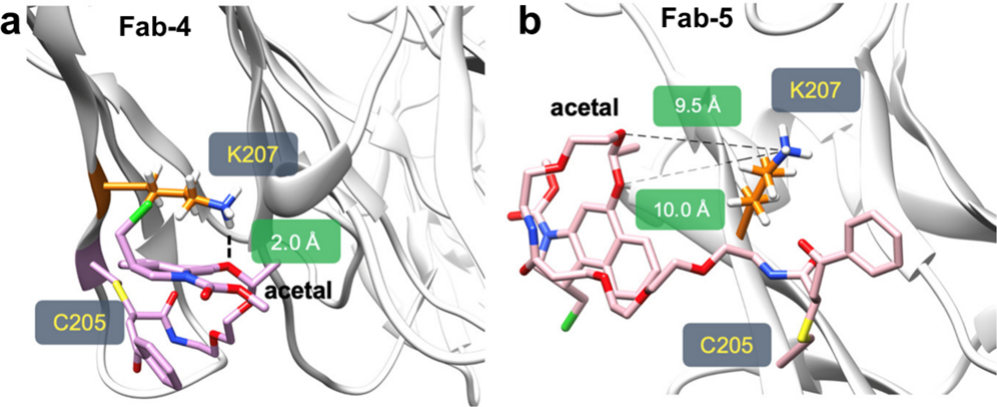
MD simulations for Fab-**4** (**a**) and Fab-**5** (**b**). Distance between acetal oxygens and K207 ammonium group is indicated in green. The R configuration was considered at both stereocenters for Fab-**5**. Figure adapted from (^2^). Copyright 2022 American Chemical Society.

While genetic mutation and synthetic design sufficiently mitigated stability issues derived from K207, we also considered how K207 might be directly harnessed for enhanced conjugate stability. As previously discussed, self-hydrolyzing maleimides containing proximal amines can stabilize bioconjugates by preventing deconjugation (Figure 2c–e),^60,61^ leading us to test if proximal K207 could similarly hydrolyze C205-maleimide conjugates. Evaluation of bioconjugates produced with maleimide **6** and Trastuzumab (WT and K207A) revealed that ∼60% more of the succinimide of Trastuzumab-**6** was hydrolyzed than that of Trastuzumab-K207A-**6** at neutral pH whereas acetal hydrolysis was unaffected (Figure 5e, f, and h). MD simulations of Fab-**6** provided further evidence for K207-mediated hydrolysis, as K207 and the succinimide were in close proximity for ∼62% of the total trajectory. These findings clearly illustrate the enhanced stability of C205-maleimide conjugates, a feature which had been previously reported but not attributed to a specific residue,^76^ and identify K207 as the causative agent.

Investigating the molecular architecture of Trastuzumab revealed K207 as an important PAB facilitator that can stabilize or destabilize bioconjugates. Factors such as linker length, linker hydrophobicity, and amino acid sequence were revealed to precisely tune bioconjugate stability due to proximity effects, features that had not been previously described in detail despite the clinical use of Trastuzumab conjugates. Future work is necessary to explore the impact of K207 on the chemistry of other C205 bioconjugates and investigate the potential correlation between the K207 proximity effects and the clinical efficacy of ADCs.

## Replicating natural PTMs using PAB

Driven by our evolving understanding of PAB strategies, we explored the use of PAB to generate homogeneous histone conjugates.^3^ Characterized by a high density of positive charge due to numerous lysine and arginine residues, histones interact directly with negatively charged DNA in nucleosomes and crucially regulate DNA replication, transcription, and repair. Post-translational acetylation of histone lysines is thought to be a key mechanism for the unwinding of DNA from nucleosomes via charge neutralization of lysines and weakened electrostatic protein–DNA interactions.^77,78^ Despite a general understanding of histone PTMs, the exact mechanisms by which specific modifications impact the nucleosome structure and further biological processes are not fully understood.

Challenges in producing and isolating homogeneous histones bearing single PTMs in large quantities have directly hindered this investigation. Elaborate bioconjugation techniques have been necessary to modify histones since traditional lysine-reactive reagents are either nonspecific, such as *N*-hydroxysuccinimide (NHS) esters, and yield heterogeneous conjugates,^79^ or ultraspecific, with their use restricted to unique residues on certain proteins.^80–82^ While native chemical ligation (NCL) and genetic code expansion can generate homogeneous histone bioconjugates, these methods are laborious and often suffer from poor yields.^83–85^ Similarly, approaches to substitute a lysine-of-interest (LOI) to cysteine and modify chemically have been effective but are unable to produce exact replicas of native protein PTMs.^86–89^ Additionally, one PAB approach achieved histone modification by anchoring an acetyl donor near a LOI with an affinity ligand, but this approach requires known, LOI-specific affinity ligands, limiting its scope.^90^

Given the apparent need for facile, regioselective histone bioconjugation strategies, our group employed PIC to develop a PAB approach for the site-specific acetylation of histone lysines.^3^ Led by Cláudia Afonso (iMM), we proposed that regioselective acetylation of an LOI could be achieved via introduction of a single proximal cysteine via mutagenesis and bioconjugation with **7** and **8** (Figure 7a). Maleimide **7** anchors to the cysteine and clicks via SPAAC to azide **8** which contains *gem*-dithioacetate, a stable acyl donor which modifies amines such as the ε-amino group of lysine residues, albeit slowly without catalysts.^91,92^ We thus proposed that *gem*-dithioacetate would be inert—provided the cysteine is first capped with **7**—until in proximity to the LOI where proximity effects could induce acetylation.

**Figure 7:**
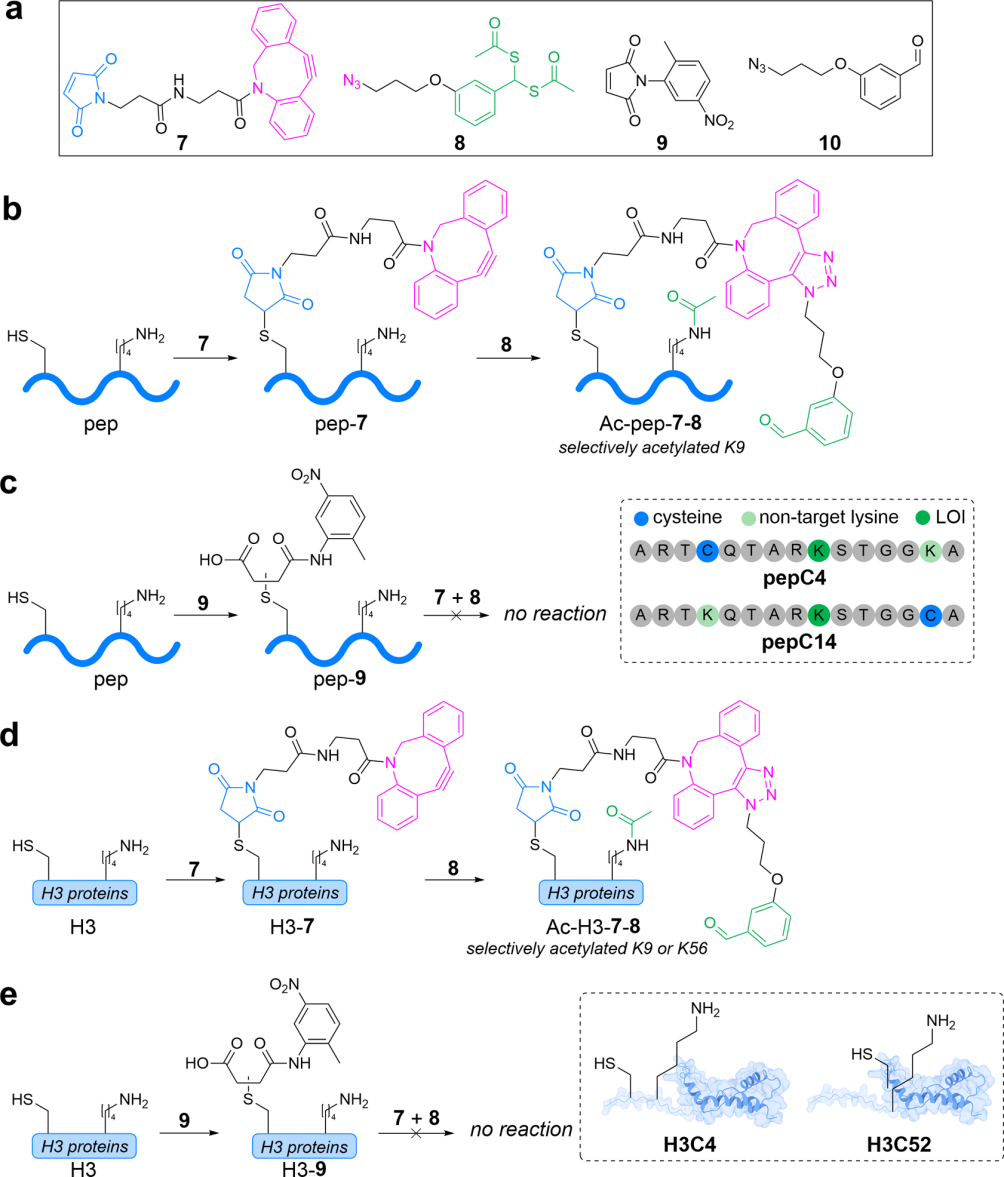
Evaluation of a novel PAB strategy for regioselective lysine acetylation on histones. (a) Molecules for PAB. (b, d) Treatment of **pepC4**/**pepC14** (b) and H3C4/H3C52 (d) with **7** and **8** sequentially yields selective lysine acetylation. (c, e) Capping cysteines of **pepC4**/**pepC14** (c) and H3C4/H3C52 (e) with **9** prevents lysine modification upon treatment with **7** and **8**. Proteins visualized in ChimeraX.

To test this approach, we generated two 15-mer peptides of the N-terminal tail of H3 which contains K9, our LOI. Acetylated-K9 (AcK9) has been studied extensively and has a known functional role in regulating transcription via recruitment of transcription factor TFIID.^93,94^ We substituted nearby lysine residues 4 and 14 for cysteine (**pepC4** and **pepC14**) and conjugated with **7** and **8** sequentially to form homogeneous AcK9 conjugates **Ac-pepC4-7-8** and **Ac**-**pepC14-7-8** (Figure 7b). MS/MS studies performed on both conjugates confirmed complete, selective acetylation of K9. Conversely, when the cysteines of **pepC4** and **pepC14** were first capped with maleimide **9** before treatment with **7** and **8**, no acetylation was observed, indicating that the acetyl donor is unreactive unless proximity is induced (Figure 7c).

To assess the generalizability of our technique, we tested this approach on two cysteine mutants of H3—H3C4 and H3C52—to target the acetylation of K9 and K56, respectively. AcK56 is another highly studied PTM known to impact transcriptional regulation.^95,96^ Gratifyingly, sequential treatment of both proteins with **7** and **8** led to regioselective acetylation of the LOIs as confirmed by mass spectrometry (Figure 7d). Neither H3C4 nor H3C52 exhibited any acetylation upon first capping the cysteine with maleimide **9**, again confirming that the proximity of the acetyl donor is required for acetylation (Figure 7e).

The biological function of these conjugates was then assessed by evaluating protein structure, binding, and ability to serve as enzymatic substrates. While our approach clearly enables regiospecific lysine acetylation, produced conjugates are ultimately mimics of H3 proteins due to the introduction and modification of a cysteine, prompting us to assess the biochemical validity of these mimics. Accordingly, H3C4-**7** and H3C52-**7** were further modified with **10** to produce H3C4-**7**-**10** and H3C52-**7**-**10** which were used as controls in these studies. Western blots revealed that all proteins and bioconjugates efficiently bound H3 antibodies, and Ac-H3C4-**7**-**8** and Ac-H3C52-**7**-**8** bound specific antibodies for their associated acetylated lysine (Figure 8a). Further analysis of H3C4 and corresponding conjugates by ELISA revealed no significant effects on binding H3 antibodies, and circular dichroism (CD) demonstrated that conjugation did not discernibly alter protein structure. Importantly, both acetylated proteins (Ac-H3C4-**7**-**8** and Ac-H3C52-**7**-**8**) could be deacetylated by Sirt3, a histone deacetylase, suggesting that these bioconjugates could be employed in further biological assays to investigate the function of PTMs (Figure 8b).

**Figure 8:**
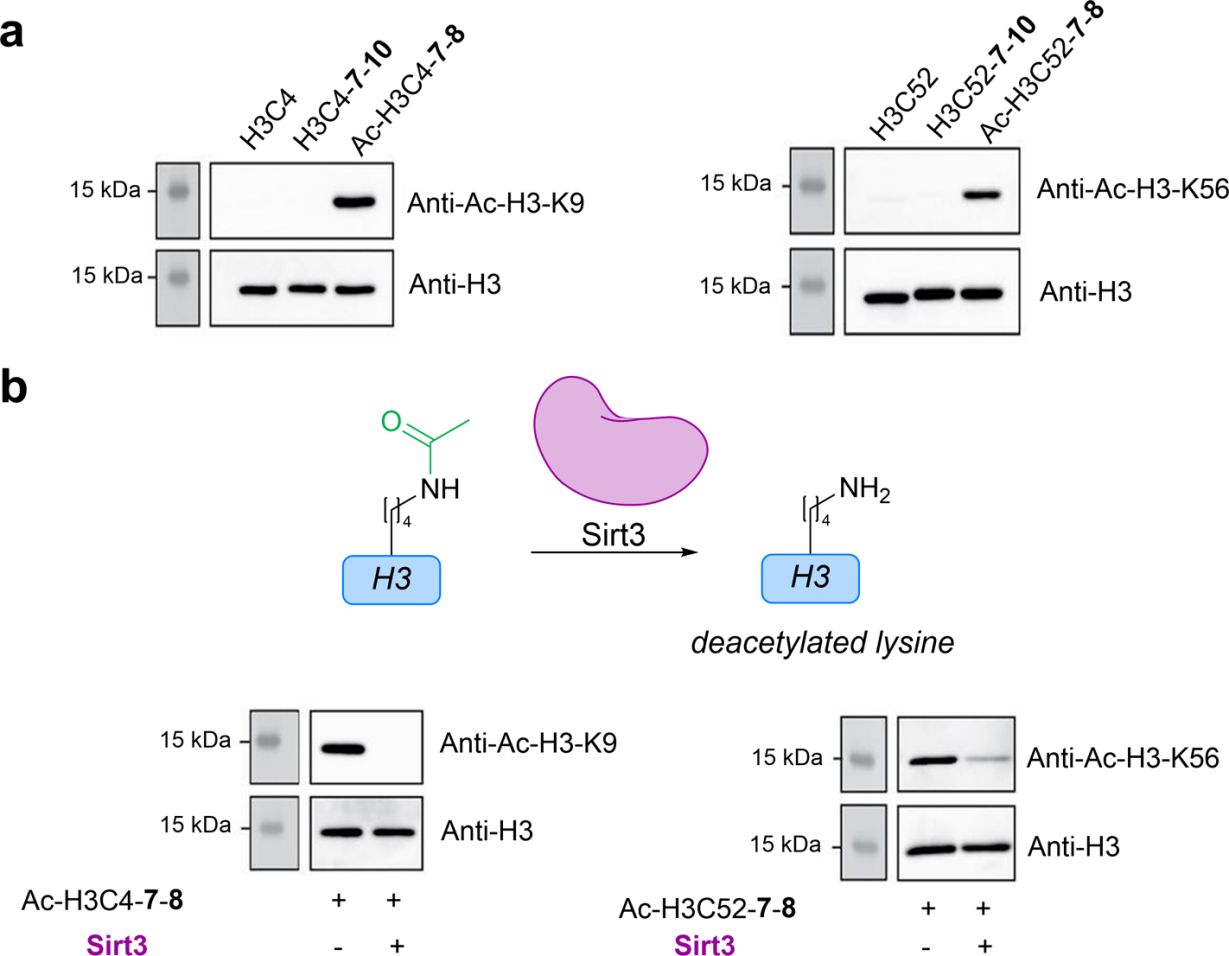
Biological characterization of acetylated H3 conjugates. (a) Western blots of conjugates from H3C4 (left) and H3C52 (right) with H3 and acetylated-H3-Lys antibodies for the corresponding lysine site. (b) Treatment of Ac-H3C4-**7**-**8** (left) and Ac-H3C52-**7**-**8** (right) with lysine deacetylase Sirt3 leads to complete or near-complete deacetylation. Figure adapted from (^3^). Copyright 2022 Wiley-VCH GmbH.

In short, this PAB strategy led to the successful development of biochemically validated, selectively acetylated H3 conjugates. Compatible with at least two lysine residues (K9 and K56), this strategy may be applicable to other residues and proteins, although further investigation of the technique’s compatibility with less accessible residues may be necessary. While this strategy requires both mutagenesis and small molecule conjugation, biochemical characterization of the conjugates revealed that this has minimal effects on histone function. For more authentic mimics, this strategy could be improved such that the cysteine modification could be reversibly removed postacetylation.

## PAB Linkers for Phage Display-Compatible Peptide Cyclization

Recently, we explored the utility of PAB for phage display-compatible peptide cyclization.^4^ Phage display is a powerful screening technique to find novel binders for therapeutic applications. Phage vectors are cloned to encode random peptides or proteins and transformed into *E. coli*, producing bacteriophage variants presenting different peptides or proteins on their surfaces. In an iterative process, the library of phage (≤10^10^ variants) is treated with an immobilized target of interest, washed, and efficient binders are eluted, amplified, and further evaluated.^97,98^

To expand the diversity of these libraries, peptide cyclization can be performed directly on phages prior to selections. Cyclic peptides have enhanced stability toward proteolysis, target specificity, and binding affinity compared to their linear counterparts.^99,100^ However, cyclization strategies that are compatible with phage display are limited, as most techniques—including disulfide cyclization, use of cysteine-reactive linkers, and ncAA incorporation—diminish phage particle viability and/or infectivity (Figure 9a).

**Figure 9:**
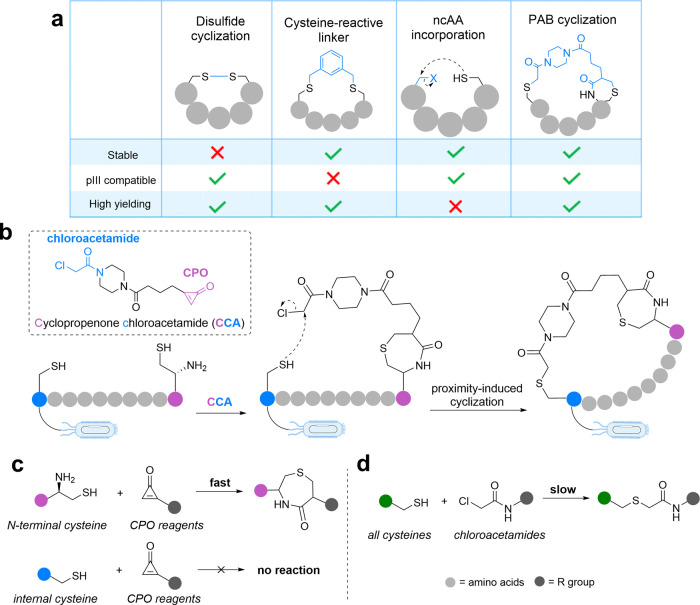
PAB technique for phage display-compatible peptide cyclization. (a) Compatibility of peptide cyclization techniques with phage display. (b) Proximity-induced peptide cyclization using CPO chloroacetamide (CCA). (c) Chemoselectivity of CPO for N-terminal cysteines. (d) Reactivity of chloroacetamides with cysteine.

Disulfide cyclization via incorporation of two cysteine residues to flank a randomized sequence spontaneously yields cyclic peptides in the oxidizing bacterial periplasm.^101^ However, this method has limited applications for therapeutic development, as these peptides rapidly linearize in reducing cellular environments *in vivo*. Thus, bifunctional cysteine-reactive linkers, such as *m*-dibromoxylene (DBX), have been utilized to generate stable cyclic peptides (Figure 9a). However, while cyclic binders with nanomolar affinity have been identified with this strategy,^102,103^ cross-reactivity of these linkers with the phage coat protein pIII (8 cysteines present) diminishes the viability and infectivity of phage particles, intrinsically limiting library size and diversity. To combat this, cysteine-free pIII mutants and the incorporation of ncAAs instead of cysteine have been assessed, both of which unfortunately suffer from diminished library size and diversity due to reduced infectivity and associated poor yields, respectively (Figure 9a).^104–106^ Thus, there remains a need for efficient, phage-compatible techniques which preserve phage particle viability and infectivity.

Led by Libby Brown (University of Cambridge), our group developed a new PAB strategy employing an asymmetric cysteine-reactive linker for phage display (Figure 9b).^4^ This linker, coined CCA (cyclopropenone chloroacetamide), includes a cyclopropenone (CPO) moiety which selectively reacts with N-terminal cysteines with rapid kinetics (*k*_2_ = 3.0 M^–1^ s^–1^) to form an irreversible seven-membered ring linkage.^107^ CPO reagents exhibit no cross-reactivity with internal cysteine residues (i.e., non-N-terminal), thereby preventing pIII conjugation (Figure 9c). The other handle of CCA, chloroacetamide, is effectively inert until in close proximity to thiols (Figure 9d).^108^ Thus, we proposed that cyclization of peptide sequences flanked by an N-terminal and internal cysteine with CCA would be achieved first via reaction of the N-terminal cysteine with CPO, followed by alkylation of the internal cysteine by chloroacetamide upon induced proximity (Figure 9b).

To test this approach, CCA was synthesized and tested with peptides in solution, wherein regioselective peptide cyclization was confirmed by LC–MS. We progressed to evaluate the efficacy of CCA for peptide cyclization on phages using a phage library to be selected for streptavidin binders. Following reduction of the peptides directly on phages with DTT and removal of linear binders, treatment with CCA successfully cyclized the peptides. Nine new cyclic binders were discovered with the strong motif CPXNX_3_PX_3_C which were notably only functional binders upon cyclization (Figure 10). Pleasingly, there were no deleterious effects on library size or diversity, and the experimental setup was simplified by the compatibility of CCA with DTT, eliminating a purification step.

**Figure 10:**
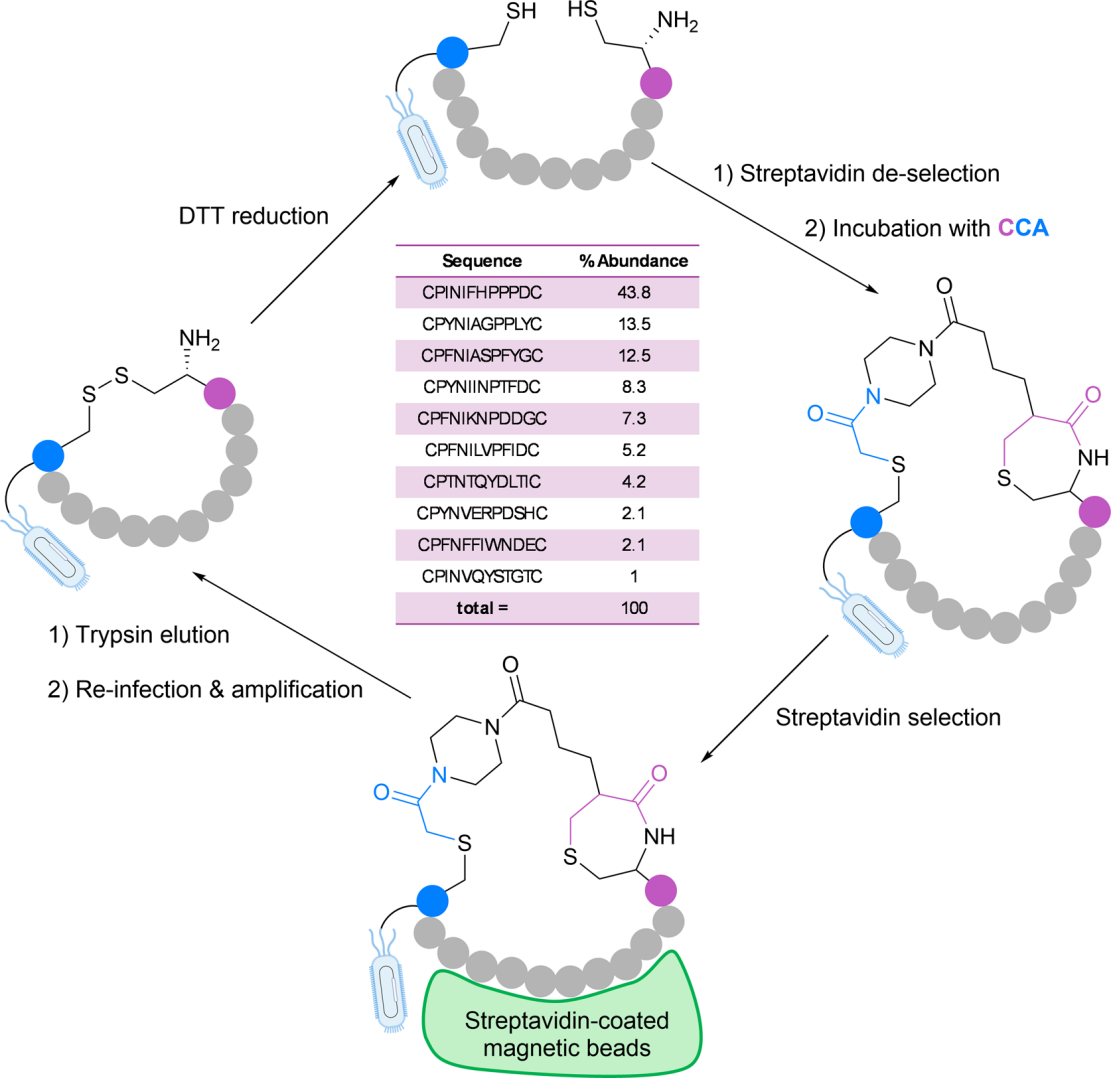
Streptavidin selection process for phage display using CCA to cyclize peptides. Figure redrawn from (^4^). Copyright 2024 Springer Nature.

Interestingly, Str7, one of the binder hits, exhibited binding only when specifically cyclized by CCA as opposed to other cysteine-reactive linkers, revealing the advantages of exploring the chemical space of the cyclization linker. In computational binding experiments, CCA-cyclized Str7 was found to bind streptavidin at a distinct site to biotin and other known streptavidin binders,^109^ illustrating the power of this technique in finding both novel binders and a novel binding site. To verify the robustness of our technique, we also tested CCA-cyclization in a phage library selected for integrin αvβ3 binders, a cell surface receptor commonly overexpressed on cancer cells. We successfully found eight cyclic peptide sequences containing a known RGD binding motif, many achieving nanomolar affinity for αvβ3.^110,111^

Together, these efforts clearly demonstrated the power of using CCA for peptide cyclization in phage display, as both phage particle infectivity and viability were preserved, and produced libraries yielded several effective cyclic binders. Although similar PAB strategies have been developed for phage display-compatible peptide cyclization, including use of a 2-cyanobenzothiazole (CBT) chloroacetamide cross-linker, our approach benefits from the superior selectivity of CPO for N-terminal cysteines whereas CBT demonstrates some reversible cross-reactivity with internal cysteines.^107,112^ Interestingly, in parallel with our work, a similar PAB approach emerged concurrently using heterobifunctional molecules containing 2-((alkylthio)­(aryl)­methylene)­malononitrile (TAMM) and chloroacetamide for phage-compatible peptide cyclization, demonstrating similar effectiveness to our approach.^51^ Ultimately, both strategies similarly harness PAB for phage display and clearly illustrate the advantages of using proximity to address established issues in chemical biology.

## Conclusions and Outlook

As a subfield of PIC, PAB functions as an advantageous approach to expand the repertoire of chemical biologists. By harnessing the latent reactivity of molecules, proximity techniques can improve upon traditional bioconjugation strategies, simplify regioselective modification of biomolecules, and enhance bioconjugate stability without the need for laborious experimental work. In this Account, we have summarized the efforts of our group to develop PAB strategies to address several key challenges in chemical biology. Findings regarding proximal lysine participation in cysteine bioconjugation revealed that a single residue of THIOMAB Trastuzumab-V205C, K207, directly modulates bioconjugate stability, greatly informing our understanding of the molecular architecture of this IgG and corresponding principles for ADC design. We further described the development of PAB techniques for regioselective lysine acetylation on histones and for phage display-compatible peptide cyclization by utilizing induced proximity to activate latently reactive functional groups.

Together, these projects reveal the utility of PAB to address three biologically relevant challenges and serve as representative models for future PAB design. Nonetheless, there remain several unexplored, potential applications for PAB including development of regioselective techniques to modify less reactive (e.g., serine, tyrosine, etc.) or less accessible residues and development of a universal PAB strategy generalizable to any biomolecule of interest, among others. We believe that exploration of PAB strategies will facilitate the bioconjugation of the most challenging targets and hope that our findings may promote further PAB efforts.

## Abbreviations

AA: amino acid
Ac: acetylated
ADCs: antibody–drug conjugates
APT1: acyl protein thioesterase 1
Asp: aspartate
BTK: Bruton’s tyrosine kinase
CCA: cyclopropenone chloroacetamide
CPO: cyclopropenone
Cys: cysteine
DBX: *m*-dibromoxylene
DMF: dimethylformamide
DNA: deoxyribonucleic acid
DTT: dithiothreitol
ELISA: enzyme-linked immunosorbent assay
ENPHASYS: enhancement of phagocytic synapses
Fab: antigen-binding fragment
FKBP: FK506-binding proteins
GSH: glutathione
H3: histone protein H3
H3C4: histone protein H3 K4C mutant
H3C52: histone protein H3 R52C mutant
His: histidine
HSA: human serum albumin
IgG: immunoglobulin G
*k*_2_: second order rate constant
KRAS^G12C^: Kirsten rat sarcoma oncogene homologue G12C mutant
LC–MS: liquid chromatography mass spectrometry
LOI: lysine of interest
Lys: lysine
LYTAC: lysosome-targeting chimera
M6P: mannose-6-phosphate
MD: molecular dynamics
mDap: *N*-maleimido-diamino propionic acid
MS/MS: tandem mass spectrometry
NaP_i_: sodium phosphate buffer
ncAA: noncanonical amino acid
NCL: native chemical ligation
NHS: *N*-hydroxysuccinimide
PAB: proximity-assisted bioconjugation
PD-1: programmed cell death protein 1
Pep: peptide
pIII: Filamentous phage protein III
PIC: proximity-induced chemistry
PINAD: proximity-induced nucleic acid degrader
POI: protein of interest
PROTAC: proteolysis-targeting chimera
PTMs: post-translational modifications
RIBOTAC: ribonuclease-targeting chimera
RNA: ribonucleic acid
RNase: ribonuclease
Ser: serine
Sirt3: sirtuin-3 deacetylase
SPAAC: strain-promoted azide–alkyne cycloaddition
STING: stimulator of interferon genes
Str7: streptavidin binder #7
TAMM: 2-((alkylthio)­(aryl)­methylene)­malononitrile
TCEP: tris­(2-carboxyethyl)­phosphine
TFIID: transcription factor II D
UV: ultraviolet
WT: wild-type
